# *COL12A1* Single Nucleotide Polymorphisms rs240736 and rs970547 Are Not Associated with Temporomandibular Joint Disc Displacement without Reduction

**DOI:** 10.3390/genes12050690

**Published:** 2021-05-05

**Authors:** Bartosz Dalewski, Katarzyna Kaczmarek, Anna Jakubowska, Kamila Szczuchniak, Łukasz Pałka, Ewa Sobolewska

**Affiliations:** 1 Department of Dental Prosthetics, Pomeranian Medical University, 70-111 Szczecin, Poland; bartosz.dalewski@pum.edu.pl (B.D.); rpsobolewski@wp.pl (E.S.); 2 Department of Genetics and Pathology, Pomeranian Medical University, 71-252 Szczecin, Poland; katarzyna.kaczm@gmail.com (K.K.); aniaj@pum.edu.pl (A.J.); 3 Independent Laboratory of Molecular Biology and Genetic Diagnostics, Pomeranian Medical University, 71-252 Szczecin, Poland; 4 Department of Dental Prosthetics, Outpatient Dental Clinic, Pomeranian Medical University, 70-111 Szczecin, Poland; 5 Private Dental Practice, 68-200 Żary, Poland; dr.lpalka@gmail.com

**Keywords:** TMJ, DDwoR, polymorphism, SNP, articular disc, TMD, disc displacement, *COL12A1*, rs970547, rs240736

## Abstract

Temporomandibular disorders (TMDs) may affect up to 25% of the population, with almost 70% of these TMD cases developing malpositioning of the disc over time in what is known as internal derangement (ID). Despite significant efforts, the molecular mechanism underlying disease progression is not yet very well known. In this study, the role of *COL12A1* rs970547 and rs240736 polymorphisms as potential genetic factors regulating ID was investigated. The study included 124 Caucasian patients of both sexes after disc displacement without reduction (DDwoR) in either one or two temporomandibular joints (TMJs), either of which meet the criteria for this condition. All patients underwent clinical examination and 3D digital imaging. The *COL12A1* rs970547 and rs240736 polymorphisms were evaluated. There were no statistically significant differences in the chi-square test between the study group and healthy controls. The examined *COL12A1* rs240736 and rs970547 polymorphisms do not contribute to DDwoR in Polish Caucasians.

## 1. Introduction

**Figure 1:**
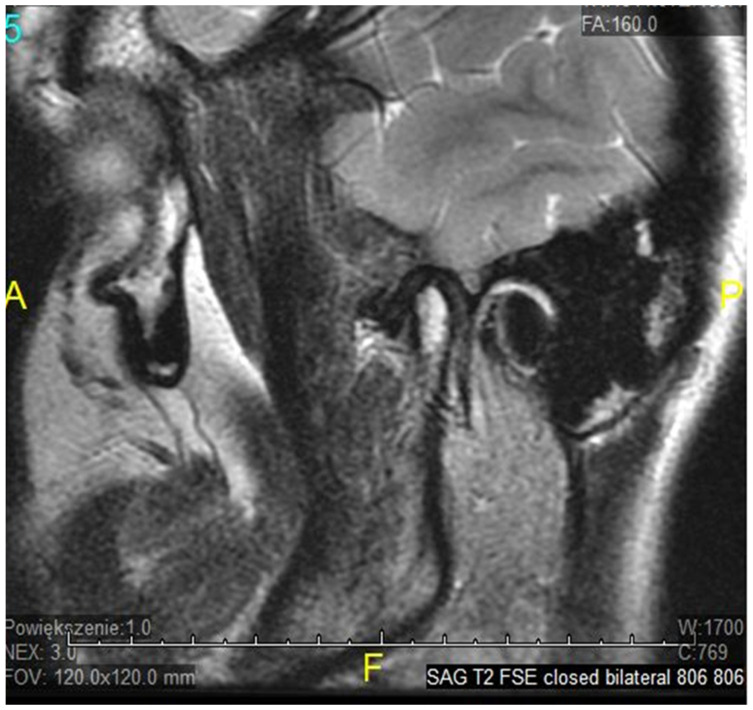
ADDwoR sagittal view—closed.

**Figure 2:**
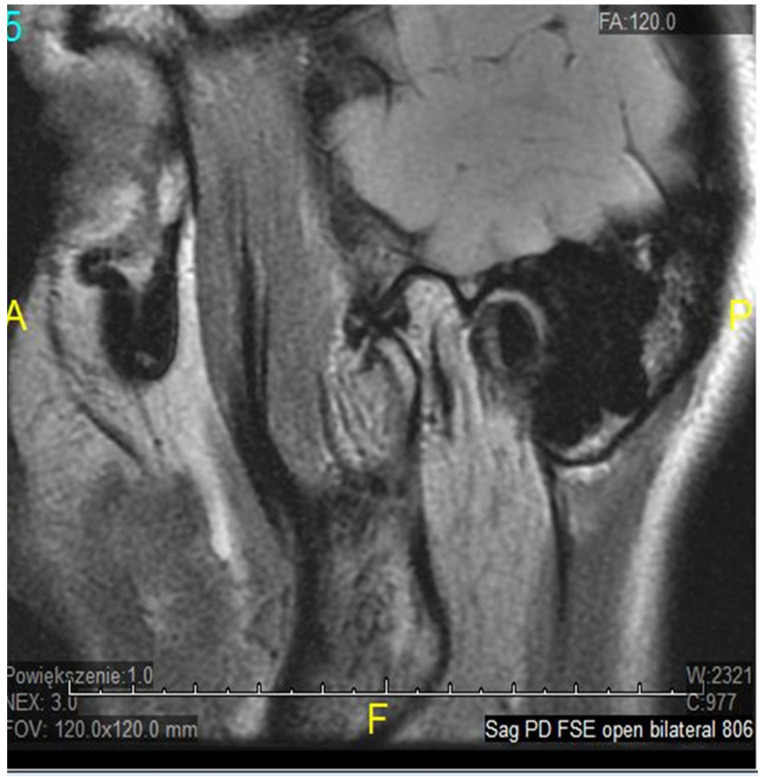
ADDwoR sagittal view—open.

**Figure 3:**
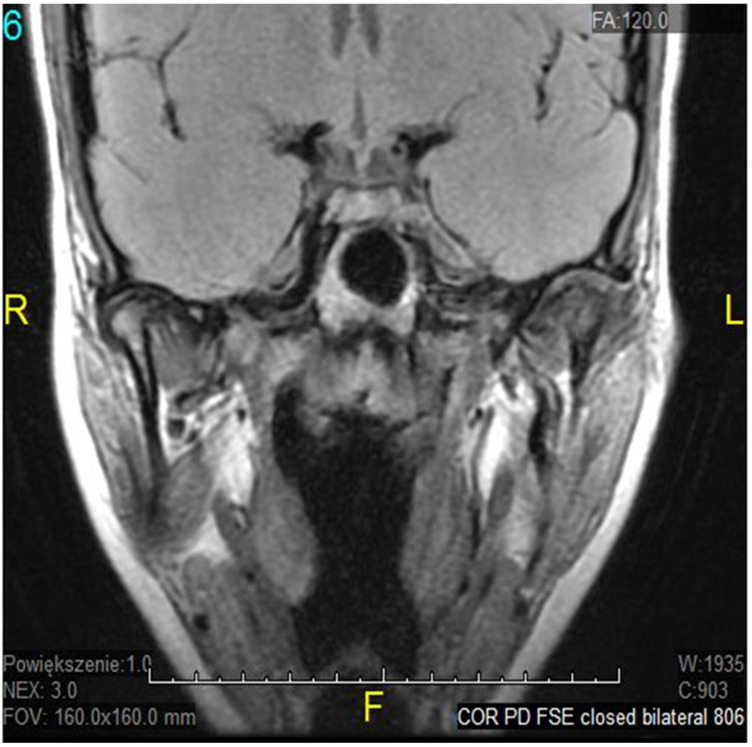
ADDwoR coronal view—closed.

The temporomandibular joint (TMJ) is a complex, bilateral structure that allows the mandible to move in three different directions. It consists of the articular surfaces of the temporal bone, the mandibular condyle, articular fibrocartilaginous disc, joint capsule, and ligaments. Articular surfaces of the condyle and the mandibular fossa are covered with fibrous (noncellular, nonvascular) cartilage, surrounded by synovial fluid, which ensures smooth and unaffected mandible movement correlated with teeth morphology throughout the chewing cycle. A fibrous capsule, covered externally by the periosteum, connects both joint surfaces and closes the synovial fluid within the joint cavity. The articular disc in the TMJ plays an inessential role in jaw kinematics and comprises the fibrocartilage, while the extracellular matrix (ECM) of this disc mostly comprises a collagenous network of mainly collagen type I and III, elastin fibers, glycosaminoglycans (GAGs), and proteoglycans [1]. So far, the expression of type I and III collagen markers in human fetal TMJ discs using immunohistochemical (IMHC) methods was confirmed [2], as they showed type I collagen presence in the posterior band of the articular disc, but type III collagen on the lower surface of the articular disc. Connective tissues exhibit heterogeneous, anisotropic, and hyperelastic activity in response to jaw movement and various occlusal loading, with collagen tissues limiting tissue expansion [3]. The ligaments in the human temporomandibular joint are partially elastic and restrict three-dimensional movement to some extent, while retrodiscal tissues play a major role in proprioception, providing sensory information about the jaw position and the force exerted by the masticatory muscles. That unique developmental composition makes the TMJ more resilient to degenerative changes over time than hyaline cartilage in other synovial joints [4]. Nevertheless, it is estimated that temporomandibular disorders (TMDs) may affect up to 25% of the population, and almost 70% of these TMD cases will develop misplacement/dystopia of the disc over time, which is called internal derangement (ID). Despite significant efforts, the molecular mechanism underlying disease progression is not yet very well known. It seems that ID strongly correlates with TMJ osteoarthritis (OA) as a symptom or a contributing factor in a later stage of TMD, affecting mostly older patients. It may occur in people of all ages, but a higher incidence was found in women in their 20s [5]. The most common types of TMJ ID are anterior or antero-mesial disc displacement (DD) with (DDwR) or without reduction (DDwoR) [6]. In DDwoR, the disc moves forwards and usually also slightly medially to the lower rest position, where it remains locked and painful upon mouth opening, as most MRI studies brought up in recent years [7,8,9]. The need for thorough evaluation and multi-section analysis in both TMJ sagittal and coronal planes has also been described [10]. The etiology is not yet fully explained, but there are a few possible reasons causing IDs of TMJ structures. According to most data, bruxism, anatomical factors, history of trauma, or generalized joint hypermobility (GJH) are considered major contributing factors [11]. A displaced TMJ disc can be reduced at an earlier stage; it is likely to transform into a non-reducing form over a few weeks. As it happens, the TMJ disc does not return to its physiological position, but becomes displaced and may prevent appropriate movement of the condyle, causing pain in the preauricular area and limitation within the mandible range of motion, affecting daily activities, as shown in Figure 1, Figure 2 and Figure 3. 

**Figure 4:**
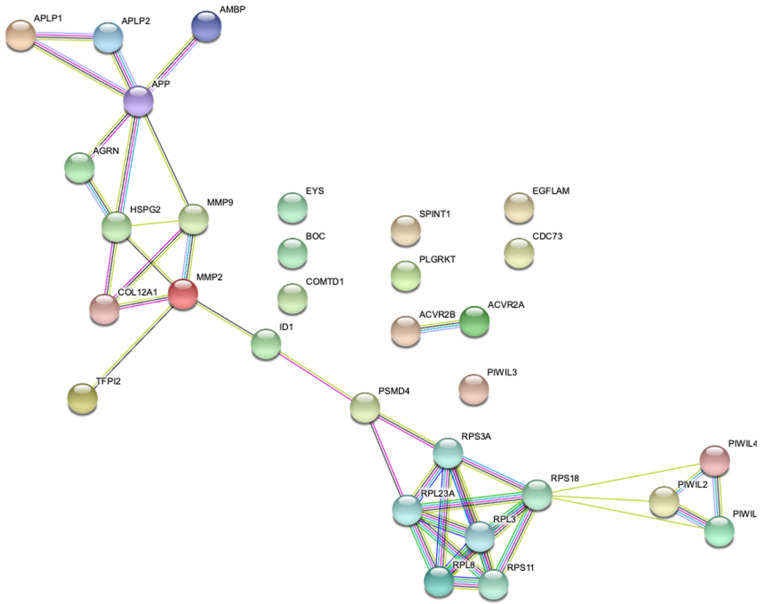
*COL12A1* possible gene–gene interactions.

Magnetic Resonance Imaging (MRI) scans showed that in cases where no treatment has been administered, the displaced TMJ disc becomes deformed [12]. It was also found that unilateral DDwoR in young people can lead to asymmetry of the mandible over time [13]. Moreover, it has been proven that the severity of asymmetry increases with time and may require orthognathic surgery later in life [14]. The TMJ disc is connected in its dorsal part with an area of richly innervated and vascularized connective tissue. This tissue is defined as the bilaminar zone (BLZ). The lower layer is mainly composed of collagen fibers. In addition, the front part of the disc is connected from the top and bottom by trailers to the joint capsule. Both front trailers are made of collagen blocks [15]. Collagen XII is a fiber-bound, single gene encoded by *COL12A1*, a member of the fibril-associated collagens with interrupted triple helices collagens (FACIT) located on human chromosome 6q12-q13. By providing unique molecular bridges between fibrils and other ECM components, the FACIT collagens tend to act as regulators of fibrillar scaffolds (Figure 1). Some partial correlation between *COL12A1* Single Nucleotide Polymorphisms (SNPs) [16] and joint disorders was previously reported; however, just one of the studies brought up the relationship between rs970547 in *COL12A1* with anterior cruciate ligament (ACL) rupture [17]. Nevertheless, more research on this subject is required in terms of other parts of the musculoskeletal system [1,2,3,4]. Furthermore, a connection between mutations in *COL12A1* and Ehlers–Danlos Syndrome (EDS) has also been demonstrated [18]; whereas a major part of EDS symptomatology is strictly connected with GJH and hyper elastic skin, TMJ posterior band ligaments, which are primarily composed of collagen fibers defined by a certain length, have also been shown to play a significant role in preventing TMJ disc displacement [19]. We hypothesized that some IDs in the TMJ might be caused by impaired collagen quality and certain *COL12A1* SNPs, yet a relationship to TMJ internal derangements might be assumed. There were some previous data published related to *COL12A1* [18] and *COL2A1* [20], however no variants of these genes in terms of DDwoR were assessed. In this study, we chose to investigate the role of *COL12A1* rs970547 and rs240736 polymorphisms as potential factors influencing genetic variability of DDwoR in Polish Caucasians. Figure 4 presents possible gene–gene interactions related to *COL12A1* according to the STRING database [21].

## 2. Materials and Methods

In this case-control study, the examined group was recruited from among patients who sought TMD treatment between 2014 and 2018 and presented to the Department of Dental Prosthetics at the Pomeranian Medical University in Szczecin, Poland. It consisted of 124 Caucasian patients, unrelated, of both sexes. Each patient had an episode of DDwoR no more than 3 months prior and signed an informed consent form before study registration. DDwoR was diagnosed according to clinical examination, diagnostic criteria of the temporomandibular disorder questionnaire (DC/TMD), and CBCT/MRI scan [22,23,24,25]. The control group comprised 126 patients with no TMD problems according to DC/TMD—they were selected from the rest of the patients treated at the outpatient dental clinic. Additional exclusion criteria for both groups were as follows: pathological tooth mobility (grade 1 or more on the Hall scale), previous experience with occlusal splint therapy, not all areas of occlusal support present, coexisting pathology or inflammation within the jaws or head and neck muscles, accompanying metabolic diseases or identified connective tissue defects.

### 2.1. SNPs Selection

In this study, we considered the genetic role of the *COL12A1* gene rs970547 and rs240736 expression as a potential cause of DDwoR expression.

DNA isolation: Genomic DNA was isolated from buccal epithelial cells using SWAB Genomic Extraction GPB Mini Kit (Genoplast Biochemical, Gdansk, Poland) in compliance with the producer’s manual.

### 2.2. Molecular Analyses

Genotyping of *COL12A1* SNPs rs970547 and rs240736 was performed by real-time PCR using TaqMan probes and analyzed using pre-designed Applied Biosystems TaqMan real-time PCR assays (Applied Biosystems, Foster City, CA, USA).

The reaction mix for each sample contained GoTaq^®^ Probe qPCR Master Mix (Promega, Madison, WI, USA), TaqMan real-time PCR assays (Applied Biosystems, Foster City, CA, USA), and nuclease-free, deionized water, strictly adhering to the manufacturer’s protocol.

The reaction mix, DNA, and no-template control (NTC) were pipetted into 384-well plates (Axygen Inc., Union City, CA, USA). Real-time PCR was performed on LightCycler^®^ 480 (Real-Time PCR System, Roche Diagnostics, Basel, Switzerland). Genotyping data were analyzed using LightCycler480 Basic Software (Version 1.5, Roche Diagnostics, Basel, Switzerland).

### 2.3. Statistical Analysis

Further on in the analysis, *COL12A1* rs970547 and rs240736 odds were calculated in respect to the most frequent combination, with respective confidence intervals of 95%. The significance of differences in the distribution of genotypes was analyzed using Pearson’s chi-square test. Logistic regression modeling was performed to analyze the association of selected SNPs with DDwoR. Data are presented as allele frequencies and odds ratio (OR) with 95% confidence interval (CI). The Mann–Whitney U test was performed to determine the age difference between the groups. *p* < 0.05 was considered to be statistically significant. Calculations were completed with MATLAB R2018b (MathWorks, Natick, MA, US, 2018).

## 3. Results

### Patient Characteristics, Odds Ratio, and Logistic Regression Analysis

The studied and acquired data were first analyzed using descriptive statistics in relation to groups. Additionally, a chi-square test was performed in order to check for independence. Table 1 describe the results of the preliminary analysis.

There is no significance in the *p*-value of the test when comparing men and women in this case-control study. The age of the case group was significantly lower than controls.

In further analyzing the polymorphisms, the odds were calculated in respect to the most frequent combination, with respective confidence intervals of 95%. A chi-squared test at 0.05 confidence level was performed in order to check for the association. The results of the odds ratio analysis are shown in Table 2. In this study, *COL12A1* markers rs970547 and rs240736 had no significant *p*-values (chi-squared), implying that there is no difference in terms of TMJ DDwoR frequency.

A multivariable logistic regression model was applied as well, but no significant model was found in the study in the case of rs970547 and rs240736. The lack of significance in the chi-square statistic vs. constant model test is shown in Table 3.

The results shown in Table 2 and Table 3 indicate that both *COL12A1* markers rs240736 and rs970547 are not associated with the odds of TMJ DDwoR.

## 4. Discussion

This is the first study of its type defining the relationship between *COL12A1* markers rs970547 and rs240736 in TMJ DDwoR. It is a part of a larger project about molecular underlying conditions influencing this issue—whereas *ESR1* rs1643821 is positively associated so far [26]. The main finding of our present investigation is that *COL12A1* markers rs240736 and rs970547 were not significant for TMJ DDwoR; therefore within our sample size, these SNPs do not contribute to the risk of articular disc displacement (ADDwR).

Differences in protein expression comparing core matrisome of 9 fetal and 7 healthy adult nucleus pulposus (NP) were previously identified with the use of proteomic and bioinformatic methods; however, collagen 12a1 protein was upregulated in the fetal NPs involved in ECM assembly pathways. By contrast, even though concentrations were low and mostly limited in the degenerated state, proteins that were usually absent in adult discs were re-expressed, with one of them showing substantially increased expression relative to stable adult discs [27]. It may suggest the involvement of collagen XII in degenerated state pathways, for instance, by acting as an inhibitor or regulator in regenerative processes. As a compensatory mechanism, these proteins may be upregulated [28]. Interestingly, mature collagen 12a1 and 14a1 are not found in the main fibrillar structure; however, the 12a1 form is thought to be involved in tail and spinal cord regeneration in certain species. It is also plays a role in the regulation and organization of collagen fibril bundles, hydration, and thickness.

Nevertheless, compelling data regarding these three types of collagen are limited and more research on their role in intervertebral disc degeneration and regenerative pathways is required [29]. Specific *COL12A1* genotypes were also previously linked to Bethlem myopathy (BM). This slowly progressing muscle disease is characterized by proximal weakness, muscle contractures, the rigidity of the spine, and skin abnormalities. One of the well-known genetic causes of BM is a dominant, or, less frequently, a recessive mutation in one of the collagen VI genes (*COL6A1*, *COL6A2,* and *COL6A3*). Hicks et al., in their studies involving 24 BM-like patients, identified novel causal variants, sequencing other genes [30]. They hypothesized that these variants may be responsible for the non-collagen VI overlapping BM phenotype in *COL12A1* in 2 families, leading to the conclusion that the disease might also be inherited in an autosomal dominant way. Similarly, Punetha et al. described dominant missense mutations in the *COL12A1* gene in mild Bethlem-like myopathy in 6 patients from 3 families. Their model patient, an 8-year old Polish girl, experienced profound hypotonia and joint hyperlaxity. They used a targeted sequencing panel to identify a potentially novel, pathogenic heterozygous missense *COL12A1* c.8329G>C (p.Gly2777Arg) variant. In addition, studies on fibroblasts revealed that the *COL12a1* protein was retained intracellularly, indicating a dominant-negative mutation. They concluded that *COL12A1* disorders seem to additionally cover a considerable part of an Ehlers–Danlos/Bethlem-like myopathy severity, and collagen XII-related conditions should become a part of a detailed examination while diagnosing patients with an overlapping phenotype that is associated with both muscle and connective tissue inherited defects [31].

Ligaments, capsules, and tendons insert into the underlying bone in a specific anatomical zone called the enthesis, a multilayered structure transmitting mechanical stress caused by movement from the tendons or ligaments onto the underlying bone. Despite the uncalcified fibrocartilage layer through which the collagen fibers of tendon or ligaments are passing, it is followed by a calcified fibrocartilage string, which subsequently inserts onto the hard tissue [32]. The irregular transition between fibrocartilage and the underlying bone increases the bond surface and adds tensile strength [33]. In chronic joint disorders, such as rotator cuff pathology, this synovio–entheseal complex is thought to play a crucial role. Type II collagen showed promising results in the Harada et al. study, indicating that rotator-cuff-derived cell sheet could facilitate cartilage regeneration and angiogenesis at the enthesis, while having superior mechanical strength to that found in the control group. However, they speculated that in the case of a rotator cuff injury, collagen-rich cell sheets might be a successful regenerative strategy for both enthesis and tendon in potential tissue engineering techniques [34]. ACL injuries were also previously associated with *COL12A1* in a Chinese population—rs970547 and rs240736 had a correlation with ACL injury frequency in Chinese men. Males with *COL12A1* rs970547A allelic gene and AA genotype were found to be more susceptible to ACL injury [35].

Collagen XII mutations have recently been linked with changes in connective tissue, with phenotypic manifestations similar to collagen VI-related myopathies. Araújo and Antunes reported a novel mutation identified in a 14-year old Caucasian girl suffering from persistent clavicle dislocation, GJH, and a small decrease of the strength of the upper limbs. Once mutations in collagen VI have been ruled out, a heterozygous missense mutation in *COL12A1*-c.8336G > A- indicates a potential link to collagen XII-related disorders in individuals with an overlapping phenotype with muscle and connective tissue defects combined [36]. Another study showed that *COL12A1* rs970547 should not be associated with the analyzed range of motion. The interaction effects between age and genotype found in the variants and range of motion measurements among studied groups were also not significant [37].

This finding is partially consistent with our research, as the *COL12A1* rs970547 group did not correlate with symptoms and their occurrence. A similar association of *COL12A* with GJH phenotypes and their phenotypic variability was emphasized and described by Jezela-Stanek et al. [38]. The *COL12A1* was associated with ACL ruptures among female participants in the study by Posthumus et al. Despite the fact that the AA genotype of the *COL12A1* AluI RFLP was substantially over-represented in female ACL patients, but not in male, it was concluded that these preliminary genetic association studies should be further investigated and, if replicated, integrated into screening models developed to identify genetically predisposed patients [39]. The *COL12A1* A9285G polymorphism was also assessed by Ficek et al. in Polish male football players. While the frequency of the G allele was lower in the cases, it was not statistically significant [40]. In a following study by Ficek et al., no association between the A9285G *COL12A1* polymorphism and ACL ruptures was found in the group of male athletes [41]. Subsequently, O’Connell et al., in a joint Polish-South African study performed on patients with surgically diagnosed ACL ruptures, scrutinized *COL12A1* rs97054, among a few other collagen SNPs. They confirmed *COL12A1* rs970547 (A/G) variants and the risk of ACL injury in females. Interestingly, *COL12A1* rs970547 was not associated with the risk of ACL injury in a larger female South African cohort. What is more, the *COL12A1* rs970547 AA genotype was significantly associated with a reduced risk of ACL ruptures in Polish female patients, consistently with earlier results [38]. These data collectively emphasize the need for investigating gene–gene interactions in the etiology of ACL ruptures and other joint–tendon pathology in multiple independent cohorts, including varying ethnicity [38,39,40,41]. Bell et al. raised the issue of different collagen genes and joint laxity, finding that the *COL12A1* AA genotype improved anterior knee laxity in comparison to the AG + GG genotypes. These findings are plausible because the rs970547 amino acid location (glycine to serine at position 3058 in the protein sequence; G3058S) is found within the NC1 functional binding domain [42], which is needed for collagen matrix organization. This NC1 domain protrudes from the main structure and serves as an interaction platform for ECMs and possibly other collagen fibers [43]. If this amino acid change alters the binding domain significantly, the altered collagen structure may be presented clinically through joint laxity and ultimately in ACL rupture [44].

The initial hypothesis of Novaretti et al. was that mRNA expression of ligament healing factors in the ACL would be higher in acute tears less than 3 months from injury than it would be in intermediate (3 to 12 months) and chronic (more than 12-month-old injuries). As it turned out, among the other genes studied, *COL12A1* expression in the ACL remnant is greater in the acute phase of healing (<3 months from injury) in comparison to chronic (>12 months old) injuries [45]. In the formed tendon, cellular structure and fibril packing were essential determinants of its biomechanical properties. Collagen XII deficiency affected the structure, relationships, and intercellular collagen communication of tenocytes. Tenocyte interaction and organization were disrupted when regulatory domains were impaired. They concluded that patients with altered *COL12A1* expression may be affected by abnormal tendon extracellular matrix composition, specifically fiber assembly [46].

## 5. Conclusions

The data gathered in this study suggest that there is no association between the *COL12A1* rs970547, rs240736 and DDwoR in Polish Caucasians. However, the lack of statistical significance in genotype and allele distribution does not rule out the possibility that the investigated polymorphisms do influence DDwoR. Pathologies associated with ligaments, joints, and tendons are complex phenomena and may be caused by a variety of proteins expressed on different chromosomes (gene–gene interactions, as shown in Figure 4). It is also plausible that interactions between these genetic factors and a number of environmental aspects (gene–environment interactions) might be crucial. Our findings may contribute to the understanding of the genetic mechanism underlying DDwoR, but further experimental studies on *COL12A1* polymorphisms, including their interaction with other genes, are needed to fully understand these processes. Furthermore, a larger sample of participants, perhaps from different ethnic backgrounds, is required to confirm our results.

## Figures and Tables

**Table 1:** Patient characteristics by group.

		***Total*** ***n = 250***		***Case*** ***n = 124***		***Control*** ***n = 126***			*p*-*Value* *
		**N**	**%**	**N**	**%**	**N**	**%**		
***Sex***	F	200	80.00	104	83.87	96	76.19		0.129
	M	50	20.00	20	16.13	30	23.81		
***Age***	<24	54	21.60	40	74.07	14	25.93		**<0.01**
	24–33	70	28.00	37	52.86	33	47.14		
	34–50	65	26.00	33	50.77	32	49.23		
	≥50	61	24.40	14	22.95	47	77.05		
***Age***		**Total**		**Case**		**Control**		***p***	
	Median (Q1–Q3)	34 (24–50)		29.5 (22–39)		39 (28–60)		<0.01	

* chi-square test.

**Table 2:** Odds ratio analysis.

		Case	Control	OR	95% CI		*p*-Value *
***COL12A1* rs970547**							
**Reference**	TT	75	76	1			0.71 **
	CC	4	2	0.4934	0.0877	2.77	
	CT	45	46	1.0088	0.5996	1.6972	
***COL12A1* rs240736**							
**Reference**	AA	72	68	1			0.157 **
	AG	36	47	1.3824	0.8006	2.3868	
	GG	15	8	0.5647	0.2251	1.4168	

* chi-square test. ** not significant.

**Table 3:** Multivariable, sex, and age-adjusted logistic regression modeling for *COL12A1* SNPs rs970547 and rs240736.

		aOR	95% CI		*p-*Value	Power
Sex	**m**	0.61	0.31	1.21	0.16	0.93
**rs970547**	**TT**	1.03	0.59	1.82	0.91	0.7
	**CC**	1.97	0.32	12.25	0.47	0.99
**rs240736**	**GG**	2.40	0.86	6.75	0.10	0.98
	**AG**	0.74	0.41	1.33	0.31	0.90
**Age**		0.96	0.94	0.97	<0.01	0.99

## Data Availability

We would not like to disclose the data, as they are sensitive according to our agreement with patients enrolled.

## Abbreviations

ACL	anterior cruciate ligament
ADD	anterior disc displacement
DDwoR	disc displacement without reduction
DDwR	disc displacement with reduction
CBCT	cone beam computed tomography
cEDS	classical type of Ehlers–Danlos syndrome
CI	confidence interval
CL	confidence level
*COL12A1*	collagen, Type XII, α 1
CTS	carpal tunnel syndrome
DC/TMD	diagnostic criteria of temporomandibular disorders
ICR	idiopathic condylar resorption
MRI	magnetic resonance imaging
n	sample size
NT	no template control
OI	osteogenesis imperfecta
OR	odds ratio
*p***-**value	level of probability
PCR	polymerase chain reaction
SNP	single nucleotide polymorphism
TMD	temporomandibular disorder
TMJ	temporomandibular joint
